# A methodological review of randomised n-of-1 trials

**DOI:** 10.1186/s13063-024-08100-1

**Published:** 2024-04-16

**Authors:** Olivia Hawksworth, Robin Chatters, Steven Julious, Andrew Cook, Katie Biggs, Kiera Solaiman, Michael C. H. Quah, Sxe Chang Cheong

**Affiliations:** 1 Sheffield Clinical Trials Research Unit (CTRU), Sheffield Centre for Health and Related Research (SCHARR), The University of Sheffield, Sheffield, UK; 2 Sheffield Centre for Health and Related Research (SCHARR), The University of Sheffield, Sheffield, UK; 3 Clinical Trials Unit, University of Southampton, Southampton, UK; 4 School of Medicine and Population Health, The University of Sheffield, Sheffield, UK

**Keywords:** n-of-1 trials, Clinical trials, Placebo, Crossover

## Abstract

**Background:**

n-of-1 trials are a type of crossover trial designed to optimise the evaluation of health technologies in individual patients. This trial design may be considered for the evaluation of health technologies in rare conditions where fewer patients are available to take part in research. This review describes the characteristics of randomised n-of-1 trials conducted over the span of 12 years, including how the n-of-1 design has been employed to study both rare and non-rare conditions.

**Methods:**

Databases and clinical trials registries were searched for articles including “n-of-1” in the title between 2011 and 2023. The reference lists of reviews identified by the searches were searched for any additional eligible articles. Randomised n-of-1 trials were selected for inclusion and data were extracted on a range of design, population, and analysis characteristics. Descriptive statistics were produced for all variables.

**Results:**

We identified 74 studies meeting our eligibility criteria, 13 of which (17.6%) were conducted in rare conditions. They were conducted in a range of clinical areas with the most common being neurological conditions (*n* = 16, 21.6%). The median (Q1, Q3) number of participants randomised was 9 (4, 20) and 12 trials (16.2%) involved a single patient only. Forty-six (62.2%) trials evaluated pharmaceutical interventions and 49 (66.2%) trials were placebo controlled. Trials had a median (Q1, Q3) of six (4, 8) periods and 61 (82.4%) compared two health technologies. Fifty-seven (77.0%) trials incorporated blinding and 32 (43.2%) had a washout period. Forty-nine trials (66.2%) used patient-reported outcome measures (PROMs) to assess the primary outcome. Trials used a range of approaches to analysis and 48 (64.9%) combined data from multiple patients. The characteristics of the n-of-1 trials conducted in rare conditions were generally consistent with those in non-rare conditions.

**Conclusions:**

n-of-1 trials are still underused and the application of the n-of-1 design for the evaluation of health technologies for rare diseases has been particularly limited. We have summarised the characteristics of randomised n-of-1 trials in rare and non-rare conditions. We hope that it can inform researchers in the design of future n-of-1 studies. Further work is required to provide guidance on specific design considerations, implementation, and statistical analysis of these studies.

**Trial registration:**

Not applicable.

**Supplementary Information:**

The online version contains supplementary material available at 10.1186/s13063-024-08100-1.

## Background

n-of-1 trials are within-patient, multi-period crossover trials designed to optimise the evaluation of health technologies in individual patients [1]. In these trials, a single patient receives one treatment for a period of time and then switches to receive another. Whilst receiving each treatment, outcomes are assessed in order that the effects of the treatments under investigation can be established. This “switching” is repeated, such that multiple measurements are obtained for each treatment. Usually, the order in which the patient receives the treatments is randomised. These n-of-1 trials can be seen as randomised controlled trials (RCTs) conducted in individual patients. Such n-of-1 trials combine methodological rigour with the investigation of treatment effects in an individual patient to enable the determination of the optimal treatment for a particular patient, enabling individualised patient care [2].

Often, n-of-1 trials are conducted as a series in which a number of patients undergo the same n-of-1 trial protocol. It is important to distinguish a series of n-of-1 trials from group-level crossover trials. For each individual n-of-1 trial in a series, the focus of the evaluation is at the level of the individual patient; however, between-patient analyses may also be conducted in order to estimate population-level treatment effects [3]. Crossover trials only produce population-level esimates of effect.

Since their introduction to the medical literature in the 1980s, n-of-1 trials have been used to evaluate quick-to-act health technologies in a range of chronic, symptomatic conditions [1, 4]. Gabler et al. reviewed the characteristics of 108 randomised n-of-1 trials published between 1986 and 2010 [5]. In this period, n-of-1 trials were primarily used to evaluate drug therapies, but also medical devices and surgical and behavioural interventions in myriad conditions. The n-of-1 trials captured in this review were varied in both their design and reporting quality and similar findings have since been reported elsewhere [6].

Whilst n-of-1 trials have typically been employed in chronic conditions such as attention deficit hyperactivity disorder (ADHD) and osteoarthritis, they could be considered to have particular utility for investigating health technologies for rare diseases [7, 8]. There is no single definition of a rare disease but the European Union considers a disease which affects less than one in 2000 people to be rare [9]. Parallel group RCTs are widely considered to be the gold standard approach to establishing whether a treatment has general efficacy; however, these trials are sometimes infeasible in rare diseases due to small and highly heterogeneous patient populations and alternative approaches may be required [7]. Investigating individual treatment responses using n-of-1 trials has been suggested as a valuable approach in the context of rare diseases [7, 10].

One review of n-of-1 trials in rare diseases has been conducted to date [11]. Focussing specifically on rare genetic neurodevelopmental disorders, Müller et al*.* identified twelve studies published between 1978 and 2017 [11]. In line with the Gabler et al*.* review, they found wide variation in design elements such as number of periods and total study length [5]. This review highlighted that, whilst being an area that may benefit from the study design, the use of n-of-1 trials has been limited in rare genetic neurodevelopmental disorders.

The present study was undertaken as part of the DIAMOND (Development of generalisable methodology for n-of-1 trials delivery for very low volume treatments) project. This project aimed to develop the methodology underpinning n-of-1 trials to improve the rigour and consistency in their use. We also aimed to enable n-of-1 trials to be employed in as many rare conditions and low-volume interventions as possible.

### Objectives

The present study had the following objectives:

1. To describe the characteristics of randomised n-of-1 trials reported since the Gabler et al*.* (2011) review
2. To identify n-of-1 trials that have been used to study rare conditions; and
3. To compare the characteristics of n-of-1 trials in rare and non-rare conditions.

## Methods

### Trial identification

We conducted searches on 5^th^ May 2021 using PubMed, EMBASE, Web of Science, the NIHR journals library and clinical trials registries (ISRCTN and ClinicalTrials.gov). We searched the term “n-of-1” in the “Title” field and applied a filter to retrieve results published since 1^st^ January 2011. The full search strategies are provided in Additional file 1. We updated the searches on 27^th^ November 2023 using the same search method, narrowing the searches to 2021 onwards. We also hand-searched the reference lists of any reviews of identified by our searches to identify any additional eligible studies (backwards citation tracking).

We screened the titles and abstracts of the resulting articles and those that did not report on an n-of-1 trial were excluded. Full-text screening for eligibility was conducted by at least one reviewer and a random 10% of those that were screened were independently checked by another reviewer. Where there was uncertainty, these were discussed with RC and SAJ.

We used the following eligibility criteria:

- The article had to report on an interventional n-of-1 trial in a human population;
- The article had to present the protocol for or the results of a study;
- The sequence of treatment episodes had to be randomised;
- Where a series of n-of-1 trials were conducted within a study, the primary analysis, and interpretation, had to be at the level of the individual;
- Abstracts were included where no full text was available, given they included sufficient information for data extraction;
- Papers in languages other than English were excluded unless an English abstract meeting criterion (e) was available; and
- Reviews of n-of-1 studies were excluded.

### Data extraction

Data were extracted by at least one reviewer into Microsoft Access (2016) and a random 10% of these were independently checked by another reviewer. Data items for extraction were adapted from Gabler et al. and are given below [5].

**Table:** 

**Study and population characteristics:** including the publication year, whether there was funding, the country where it was undertaken, whether it was a protocol or presented the results of the study, target sample size (for protocols only), number of patients randomised, number of patients completing (as appropriate) and age group of participants. **Design characteristics:** including the intervention type, health/disease area, whether it was rare condition (defined as one affecting less than one in 2000 people in the general population), number of health technologies evaluated, number of periods, period length, number of crossovers, if there was a washout period, length of washout, type of comparator, blinding, total duration of the study, type of primary outcome measurement and whether the primary outcome was measured multiple times per period. **Analysis characteristics:** including the definition of response to treatment, type of analysis, whether the study combined the results from multiple patients, whether numerical results were reported and whether the study quoted *P*-values.	

The full dataset is given in an additional file (see Additional file 2).

### Data analysis

Descriptive statistics were produced for all variables. Discrete variables were expressed as counts and percentages, and continuous variables were expressed as medians with the first and third quartiles. Missing data were coded as “not reported” for discrete variables and excluded from the analysis of continuous variables.

## Results

**Fig. 1:**
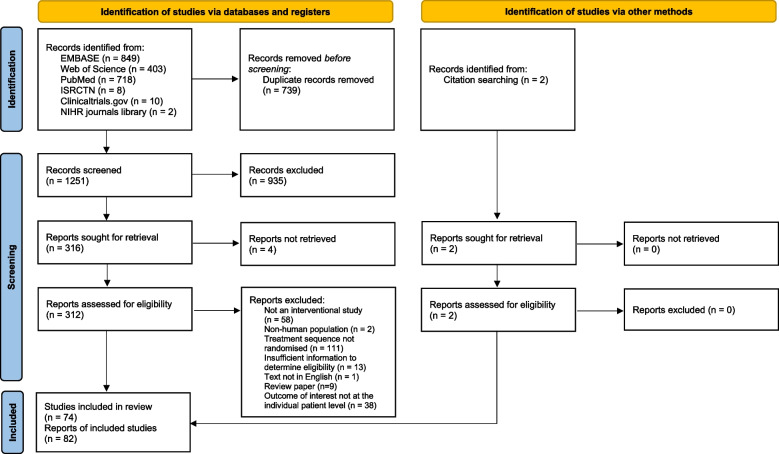
PRISMA flowchart detailing selection of studies for inclusion

Figure 1 gives a breakdown of the studies identified [12]. Searches identified 1990 records and, after the removal of duplicates, 1251 unique records were screened. Of these, 316 were sought for retrieval and 82 reports of 74 studies were found to be eligible for inclusion. Bibliographic citations for all included studies are provided in Additional file 3.

### Study and population characteristics

Table 1 gives the study and population characteristics. Thirteen studies (17.6%) were identified as being conducted in rare conditions. Nineteen (25.7%) of the included articles were protocols for n-of-1 studies and the remainder (*n* = 55, 74.3%) reported on the results of completed studies. Fifty-four (25.7%) studies reported funding, 10 (13.5%) reported receiving no funding, and 10 (13.5%) did not report on whether they had received funding.

**Table 1:** Study and population characteristics of included studies

Study and population characteristics	Rare (*n* = 13)	Non-rare (*n* = 61)	Total (*n* = 74)
*Protocol only, n (%)*			
Yes	5 (38.5)	14 (23.0)	19 (25.7)
No	8 (61.5)	47 (77.0)	55 (74.3)
*Funding, n (%)*			
Yes	9 (69.2)	45 (73.8)	54 (73.0)
No	1 (7.7)	9 (14.8)	10 (13.5)
Not given	3 (23.1)	7 (11.5)	10 (13.5)
*Country, n (%)*			
Australia	0 (0.0)	8 (13.1)	8 (10.8)
Brazil	0 (0.0)	2 (3.3)	2 (2.7)
Canada	0 (0.0)	7 (11.5)	7 (9.5)
China	2 (15.4)	8 (13.1)	10 (13.5)
Colombia	0 (0.0)	1 (1.6)	1 (1.4)
Finland	0 (0.0)	2 (3.3)	2 (2.7)
France	1 (7.7)	3 (4.9)	4 (5.4)
Germany	0 (0.0)	3 (4.9)	3 (4.1)
Italy	0 (0.0)	2 (3.3)	2 (2.7)
Korea	0 (0.0)	1 (1.6)	1 (1.4)
Netherlands	8 (61.5)	1 (1.6)	9 (12.2)
Norway	1 (7.7)	1 (1.6)	2 (2.7)
Portugal	0 (0.0)	1 (1.6)	1 (1.4)
Sweden	0 (0.0)	1 (1.6)	1 (1.4)
UK	0 (0.0)	6 (9.8)	6 (8.1)
USA	1 (7.7)	14 (23.0)	15 (20.3)
*Health/disease area, n (%)*			
Cancer	0 (0.0)	2 (3.3)	2 (2.7)
Cardiovascular	0 (0.0)	6 (9.8)	6 (8.1)
Chronic pain	0 (0.0)	4 (6.6)	4 (5.4)
Diabetes	1 (7.7)	0 (0.0)	1 (1.4)
Gastrointestinal	0 (0.0)	4 (6.6)	4 (5.4)
Genetic neurodevelopmental	1 (7.7)	1 (1.6)	2 (2.7)
Genitourinary	1 (7.7)	1 (1.6)	2 (2.7)
Headache	0 (0.0)	1 (1.6)	1 (1.4)
High cholesterol	0 (0.0)	1 (1.6)	1 (1.4)
Hyperkalaemic periodic paralysis	1 (7.7)	0 (0.0)	1 (1.4)
Musculoskeletal	1 (7.7)	2 (3.3)	3 (4.1)
Neurological	4 (30.8)	12 (19.7)	16 (21.6)
Neuropsychiatric	1 (7.7)	2 (3.3)	3 (4.1)
Non-specific	0 (0.0)	6 (9.8)	6 (8.1)
Physical activity	0 (0.0)	5 (8.2)	5 (6.8)
Prosthetics	0 (0.0)	1 (1.6)	1 (1.4)
Psychiatric	0 (0.0)	3 (4.9)	3 (4.1)
Pulmonary/respiratory	1 (7.7)	7 (11.5)	8 (10.8)
Renal	2 (15.4)	2 (3.3)	4 (5.4)
Thyroid disorder	0 (0.0)	1 (1.6)	1 (1.4)
*Number of participants randomised, median (Q1, Q3)*	6 (4, 9)	10 (3, 20)	9 (4, 20)
*Number of participants completing, median (Q1, Q3)*	5 (3, 6)	8 (3, 15)	7 (3, 14)
*Age group of included participants, n (%)*			
Children only (< 18 years)	0 (0.0)	8 (13.1)	8 (10.8)
Adults only	9 (69.2)	51 (83.6)	60 (81.1)
Children and adults	2 (15.4)	2 (3.3)	4 (5.4)
Not reported	2 (15.4)	0 (0.0)	2 (2.7)
*Target sample size (protocols only), median (Q1, Q3)*	6 (5, 18)	40 (20, 47)	20 (10, 43)

*Categories are not mutually exclusive

Studies were conducted in a range of countries including Australia (*n* = 9, 10.8%), Canada (*n* = 7, 9.5%), China (*n* = 10,13.5%), Netherlands (*n* = 9, 12.2%), United Kingdom (*n* = 6, 8.1%) and United States of America (*n* = 15, 20.3%).

They spanned a range of health and disease areas, with the most common being neurological conditions (*n* = 16, 21.6%). Six studies (8.1%) were not conducted in a specific disease area, e.g. blood transfusion-dependent patients.

Most of the studies included adult participants only (*n* = 60, 81.1%). Eight studies (10.8%) only included children (aged < 18 years old). Four studies (5.4%) included both adults and children. Studies were mostly of series of n-of-1 trials; 12 (16.2%) studies included a single participant only. The median (Q1, Q3) number of participants randomised was nine (4, 20) and the number of participants completing the studies was seven (3, 14). For the included protocols, the median (Q1, Q3) target sample size was 20 (10, 43).

The study and population characteristics were similar for studies conducted in rare and non-rare conditions, except for the number of participants involved (both randomised and completing) in the studies which was lower for the studies in rare conditions. A similar difference is seen in the target sample sizes stated in the study protocols.

### Design characteristics

Table 2 gives the design characteristics for the studies. The types of health technologies evaluated were: pharmaceutical (*n* = 46, 62.2%); nutritional (*n* = 4, 5.4%); medical device (*n* = 6, 8.1%); behavioural (*n* = 7, 9.5%); surgical (*n* = 1, 1.4%) and other types (*n* = 10, 13.5%). The comparators used were: placebos (*n* = 49, 66.2%); active treatments (*n* = 21, 28.4) and no intervention (*n* = 10, 13.4%). Studies most commonly compared two health technologies (*n* = 61, 82.4%). Nine studies (12.2%) compared three health technologies, three studies compared four health technologies (4.1%), and one study compared five health technologies (1.4%).

**Table 2:** Design characteristics of included studies

Design characteristics	Rare (*n* = 13)	Non-rare (*n* = 61)	Total (*n* = 74)
*Intervention type, n (%)*			
Pharmaceutical	10 (76.9)	36 (59.0)	46 (62.2)
Nutrition	1 (7.7)	3 (4.9)	4 (5.4)
Medical device	1 (7.7)	5 (8.2)	6 (8.1)
Behavioural	0 (0.0)	7 (11.5)	7 (9.5)
Surgical	0 (0.0)	1 (1.6)	1 (1.4)
Other	1 (7.7)	9 (14.8)	10 (13.5)
Comparator type, n (%)*			
Placebo	10 (76.9)	39 (63.9)	49 (66.2)
Active treatment	3 (23.1)	18 (29.5)	21 (28.4)
No intervention	0 (0.0)	10 (16.4)	10 (13.5)
Number of health technologies, n (%)**			
2	12 (92.2)	49 (80.3)	61 (82.4)
3	1 (7.7)	8 (13.1)	9 (12.2)
4	0 (0.0)	3 (4.9)	3 (4.1)
5	0 (0.0)	1 (1.6)	1 (1.4)
*Number of periods, median (Q1, Q3)*	6 (4, 6)	6 (4, 9)	6 (4, 8)
*Period length (days), median (Q1, Q3)*	28 (11, 35)	14 (4, 21)	14 (5, 28)
*Blinding, n (%)*			
Yes	12 (92.3)	45 (73.8)	57 (77.0)
No	1 (7.7)	16 (26.2)	17 (23.0)
*Washout period, n (%)*			
Yes	8 (61.5)	24 (39.3)	32 (43.2)
No	5 (38.5)	37 (60.7)	42 (56.8)
*Washout length (days), median (Q1, Q3)*	7 (2, 7)	7 (3, 14)	7 (2, 14)
*Total study duration (days), median (Q1, Q3)*	119 (46, 203)	77 (42, 126)	77 (42, 168)
*Primary outcome measurement, n (%)*			
Behavioural test	1 (7.7)	3 (4.9)	4 (5.4)
Clinical assessment	1 (7.7)	2 (3.3)	3 (4.1)
Lab parameter	1 (7.7)	0 (0.0)	1 (1.4)
Physiological parameter	1 (7.7)	10 (16.4)	11 (14.9)
Patient-reported outcome	9 (69.2)	40 (65.6)	49 (66.2)
Physical activity	0 (0.0)	5 (8.2)	5 (6.8)
Lab parameter and patient-reported outcome	0 (0.0)	1 (1.6)	1 (1.4)
*Primary outcome measured multiple times per period, n (%)*			
Yes	6 (46.2)	34 (55.7)	40 (54.1)
No	7 (53.8)	25 (41.0)	32 (43.2)
Not reported	0 (0.0)	2 (3.3)	2 (2.7)

*Categories are not mutually exclusive and some studies had more than one comparator: three had placebo and an active comparator, two had an active comparator and no intervention comparator, and one had placebo and a no intervention comparator. ** Includes placebo and no intervention

The median (Q1, Q3) number of periods was six (4, 8). The median (Q1, Q3) period length was 14 days (5, 28). The majority of studies blinded participants to treatment allocation (*n* = 57, 77.0%).

Washout periods were used in 32 studies (43.2%). The median (Q1, Q3) washout length was 7 days (2, 14). The median (Q1, Q3) total study duration was 77 days (42, 168).

A range of primary outcome measures were used: patient-reported outcome measure (PROM, *n* = 49, 66.2%); physiological parameter (*n* = 11, 14.9%); lab parameter (*n* = 1, 1.4%); clinical assessment (*n* = 3, 4.1%); behavioural test (*n* = 4, 5.4%); physical activity (*n* = 5, 6.8%); lab parameter and PROM (*n* = 1, 1.4%). The primary outcome was measured once per period in 32 studies (43.2%), multiple times per period in 40 studies (54.1%) and not reported in two studies (2.7%).

The design characteristics were mostly similar for studies in rare and non-rare conditions, however there were a few differences. None of the studies in rare conditions used a “no intervention” comparator, compared to ten (16.4%) of the studies in non-rare diseases. A higher incidence of blinding was identified in the studies in rare conditions (*n* = 12, 92.3%) compared to those in non-rare conditions (*n* = 45, 73.8%). The median period length was longer for those studies in rare conditions than for those in non-rare conditions (28 days compared to 14 days). Similarly, the median study length was longer for those studies in rare conditions than for those in non-rare conditions (119 days compared to 77 days). Washout periods were more commonly used in the studies in rare conditions (*n* = 8, 61.5%) compared to studies in non-rare conditions (*n* = 24, 39.3%).

### Analysis characteristics

Table 3 gives the analysis characteristics for the study. The included studies used a range of approaches for their analysis. Formal statistical approaches included: regression models (*n* = 17, 23.0%); *t*-tests (*n* = 17, 24.0%); Bayesian approaches (*n* = 11, 14.9%) and non-parametric analyses (*n* = 9, 12.2%). Graphs or visual inspection of data were used in four studies (5.4%). Ten studies stated they did not use formal analysis methods (15.4%) and methods of analysis were not reported in six studies (11.5%).

**Table 3:** Analysis characteristics of included studies

Analysis characteristics	Rare (*n* = 13)	Non-rare (*n* = 61)	Total (*n* = 74)
*Definition of treatment response, n (%)*			
*P* value < 0.05	4 (30.8)	11 (18.0)	15 (20.2)
*P* value < 0.1	0 (0.0)	1 (1.6)	1 (1.4)
Statistical significance (*p* value unspecified)	0 (0.0)	3 (4.9)	3 (4.1)
Other statistical definition	1 (7.7)	2 (3.3)	3 (4.1)
Specified numeric change in outcome	1 (7.7)	10 (16.4)	11 (14.9)
Favourable response in all cycles	0 (0.0)	4 (6.6)	4 (5.4)
Favourable response in set number of cycles	0 (0.0)	1 (1.6)	1 (1.4)
Specified symptom change	0 (0.0)	1 (1.6)	1 (1.4)
Not given	7 (53.8)	28 (45.9)	35 (47.3)
Analysis approach, n (%)*			
Bayesian model	4 (30.8)	7 (11.5)	11 (14.9)
Non-parametric test	1 (7.7)	8 (13.1)	9 (12.2)
*t*-test	1 (7.7)	16 (26.2)	17 (23.0)
Graph or visual examination	0 (0.0)	4 (6.6)	4 (5.4)
Regression model	4 (30.8)	13 (21.3)	17 (23.0)
No formal statistical analysis	2 (15.4)	8 (13.1)	10 (13.5)
Linear mixed model	0 (0.0)	3 (4.9)	3 (4.1)
Random subjects effects model	0 (0.0)	1 (1.6)	1 (1.4)
Randomisation test	0 (0.0)	1 (1.6)	1 (1.4)
Time series analysis	0 (0.0)	1 (1.6)	1 (1.4)
Not given	1 (7.7)	7 (11.5)	8 (10.8)
*Individual data pooled, n (%)*			
Yes	6 (46.2)	26 (42.6)	32 (43.2)
No	2 (15.4)	12 (19.7)	14 (18.9)
Planned	4 (30.8)	12 (19.7)	16 (21.6)
Not applicable (single patient)	1 (7.7)	11 (18.0)	12 (16.2)
*Numerical results reported, n (%)*			
Yes	8 (61.5)	31 (50.8)	39 (52.7)
No	0 (0.0)	16 (26.2)	16 (21.6)
Not applicable (protocol only)	5 (38.5)	14 (23.0)	19 (25.7)
*P values reported, n (%)*			
Yes	3 (23.1)	24 (39.3)	27 (36.5)
No	5 (38.5)	23 (37.7)	28 (37.8)
Not applicable (protocol only)	5 (38.5)	14 (23.0)	19 (25.7)

*Categories are not mutually exclusive

The studies defined “responders” to treatment in a variety of ways. The most common definitions were statistical significance with a *p* value < 0.05 (*n* = 15, 20.2%) and a specified numeric change in the outcome measure (*n* = 11, 14.9%). Almost half of the studies did not define what they would consider a responder (denoted as “Not given” in Table 3, *n* = 35, 47.3%).

The majority of studies combined the data from multiple n-of-1 trials or, in the case of study protocols, planned to do so (*n* = 48, 64.9%). There were 14 studies which involved multiple participants but did not combine the data across n-of-1 trials (18.9%). Combining data from multiple n-of-1 trials was not possible in trials including only one patient (*n* = 12, 16.2%).

Numerical results for individual participants were reported in 39 studies (52.7%) and *P*-values were reported in 27 studies (36.5%).

The analysis characteristics were similar for the studies in rare and non-rare conditions.

## Discussion

We undertook a review of the characteristics of randomised n-of-1 trials published between 2011 and 2023. We identified 74 such trials. Most commonly the trials evaluated pharmaceutical interventions; however, we found examples evaluating a range of other intervention types including nutritional and behavioural interventions as well as medical devices. n-of-1 trials had a median of six periods with a median of 14 days duration. Most of the studies were blinded and used PROMs to measure the primary outcome. We identified 13 n-of-1 trials conducted in rare conditions, the design of which showed similar characteristics to those in non-rare conditions.

A strength of our study is that it is a comprehensive review of randomised n-of-1 trials conducted over a 12-year period. It is the first to identify a subset of n-of-1 trials conducted in rare conditions.

The study does have some limitations, however. The first limitation is that screening for inclusion and data extraction were predominantly conducted by one reviewer which may have resulted in bias and errors in study selection. However a subset of decisions were reviewed by a second reviewer, and no disagreements occurred. A further limitation may be the search strategy as we only searched the term “n-of-1”. Other work suggests that there is variability in the language used when reporting on these studies. In their review of n-of-1 studies in rare neurodevelopmental disorders, Müller et al*.* found that only two of their 12 included studies identified themselves as an n-of-1 trial [11]. However, we supplemented our database searches with a citation-tracking method to increase the sensitivity of the overall search strategy. The eligibility criteria we applied mean that the review may underestimate the total number of n-of-1 trials that have been conducted over this period. Another application of the n-of-1 design is in parallel studies which randomise participants to either undergo an n-of-1 trial or usual care. Such studies were outside the scope of the current review and have been reviewed elsewhere [13]. An additional limitation is that any findings about n-of-1 trials in rare conditions may be biased by the small number of these which have been conducted and, as a result, any conclusions drawn on the basis of these studies should be interpreted with caution. Lastly, we did not identify the protocols for studies for which the results were published. Consideration of these might have provided additional insights.

Our study updated Gabler et al.’s review of n-of-1 trials [5]. The characteristics of n-of-1 trials identified in our review broadly matched those in the Gabler et al. review. Both reviews found the most common intervention type to be pharmaceutical interventions, although Gabler et al*.* found these to represent a higher proportion of their studies compared to our review (94% compared to 62.2%). Gabler et al. also found a higher occurrence of blinding (98% compared to 77.0%). We expect that this is a consequence of our review identifying more behavioural, nutritional, and ‘other’ intervention types, which are typically more difficult to blind than pharmaceutical interventions. Both studies found PROMs to be the most common approach to measuring the primary outcome and found a similar proportion of studies which used a washout period (38% in Gabler et al*.* and 43.2% in our review). Both reviews found a median of two health technologies were compared within the trials and both found wide variation in the total length of the studies albeit with a similar median (70 days in Gabler et al. and 77 days in our review). Both reviews identified a range of approaches to analysis. There were some differences in the data extracted in the two reviews. Whilst Gabler et al*.* captured additional details on the analysis of the trials and treatment change following the n-of-1 trial, our review focussed on identifying a subset of studies conducted in rare conditions.

Müller et al*.* identified 12 n-of-1 trials in rare neurodevelopmental disorders, seven of which were randomised [11]. Two of the studies that were included in Müller et al*.*’s review were also included in our review. The characteristics of these studies differed somewhat from those identified in our review. Most of these trials evaluated dietary interventions of some kind (the authors distinguished between dietary therapies and dietary supplements) and only a quarter of the trials evaluated pharmaceutical interventions. Just one of the 12 trials used a washout period. The lower occurrence of washout periods compared to our study might be a consequence of there being fewer studies of pharmaceutical interventions identified by Müller et al.. It seems that n-of-1 trials in rare neurodevelopmental disorders differ from n-of-1 trials more generally and this may be due to specific constraints of the clinical area.

The findings of the review extend our knowledge of the ways n-of-1 trials have been designed over this period. Whilst n-of-1 trials have been suggested as a useful approach for evaluating health technologies in rare diseases, we only identified 13 studies of this kind [7, 8]. Further guidance may be needed to support the implementation of the n-of-1 trials, particularly in rare diseases. Methodological advances in this area may have the potential to improve outcomes for patients and the present review serves as a starting point for work to develop guidance for the design and implementation of n-of-1 trials.

We found that the characteristics of n-of-1 trials in rare conditions were generally similar to those in non-rare conditions. Whilst the number of studies is small, we tentatively suggest that the design of such trials does not need to be different in rare disease areas and researchers can use the larger body of literature on n-of-1 trials in non-rare conditions to inform the design of n-of-1 trials in rare conditions. We did identify some differences in design characteristics, including period length and the appropriate comparator; however, these choices are more likely to depend on the specific intervention being evaluated rather than whether the condition is rare. It is likely that there may be different practical and ethical considerations when designing n-of-1 trials in rare diseases, including those related to the availability of patients.

In addition, we found that around a third of studies calculated *P*-values as part of their analysis. It could be argued that *P*-values should be interpreted with caution in n-of-1 studies as a non-significant *P*-value does not necessarily mean there is no clinically meaningful effect. Point estimates and confidence intervals describing a range of plausible effects may be more informative in such studies.

We hope that the findings of our review can inform researchers who are looking to design an n-of-1 trial. n-of-1 trials are of particular value in rare diseases; however, our review identified few examples of n-of-1 trials being used in such diseases. Consideration of the way that n-of-1 trials have previously been designed, alongside further methodological and statistical work, can inform the design and implementation of future n-of-1 trials. The n-of-1 trials identified in the review had a median of six periods, which may represent a trade-off between precision (favouring more periods) and feasibility (favouring fewer periods). This decision would also be influenced by the length of the study periods and the length of washout needed. Similarly, the median number of health technologies compared in these studies was two. Whilst there is precedent for comparing a larger number of health technologies within these studies (we identified 13 studies), it might be the case that it is preferable to compare two health technologies. n-of-1 trials have typically been blinded where feasible. Barriers to blinding are more prevalent for behavioural interventions than for pharmaceutical interventions. Whilst blinding is preferred, it is still possible to conduct an open-label n-of-1 trial.

Future studies should focus on the statistical methods used in n-of-1 trials, including whether external data are used in their analysis and interpretation. Senn has written a tutorial article on the analysis of continuous data from n-of-1 trials using frequentist approaches [14]. Using a case study from Kaplan et al*.* [15], Schmid and Yang [16] have described Bayesian approaches for the analysis of n-of-1 trials. They highlight how, due to the small amount of data being collected in n-of-1 trials, the prior can have a large influence on the posterior. Given the influence of the prior in n-of-1 studies, further work is required on the method of elicitation. Further work establishing any relationships between the methods used and the findings of n-of-1 trials might be beneficial in optimising the design of such studies. Further work is also required to provide guidance on specific design considerations and the implementation of these studies as well as to identify any additional considerations for the use of n-of-1 trials in rare conditions.

### Supplementary Information

**Additional file 1.** Search Strategy. This file contains the search strategy used in the review

**Additional file 2.** Extracted Data. This file contains the extracted data from the review

**Additional file 3.** Included Studies. This file contains references to the studies included in the review

## Data Availability

All data generated or analysed during this study are included in this published article and its additional files.

## Abbreviations

DIAMOND: Development of generalisable methodology for n-of-1 trials delivery for very low volume treatments
NIHR: National Institute for Health and Care Research
PROMs: Patient-reported outcome measures
