# Supplementary material for: A methodological review of randomised n-of-1 trials
Source: Trials. 2024 Apr 16;25:263. doi: 10.1186/s13063-024-08100-1 (PMC11020886; doi:10.1186/s13063-024-08100-1)
Supplement: Supplementary file 1 — Additional file 1. Search Strategy. This file contains the search strategy used in the review [file 13063_2024_8100_MOESM1_ESM.docx]

**Search strategy**

We initially conducted searches on 5^th^ May 2021 and updated these searches on 27^th^ November 2023.

**PubMed**

(“n-of-1” [Title/Abstract] AND (2011:2021 [pdat])

(“n-of-1” [Title/Abstract] AND (2021:2023 [pdat])

**EMBASE**

1. “n-of-1”.m_titl.
2. limit 1 to (human and english language and yr = “2011-2021”)
3. “n-of-1”.m_titl.
4. limit 1 to (human and english language and yr = “2021 – 2023”)

**Web of Science**

TI = (n-of-1)

Timespan: 2011-01-01 to 2021-05-05 (Publication date)

TI = (n-of-1)

Timespan: 2021-05-05 to 2023-11-27 (Publication date)

**NIHR Journals Library**

“n-of-1”

Filters: 2011 - 2021

“n-of-1”

Filters: 2021 – 2023

**ISRCTN**

Text search: “n-of-1”

2011-2021

Text search: “n-of-1”

2021-2023

**ClinicalTrials.gov**

Other terms: “n-of-1”

Filters: completed studies, studies with results

2011-2021

Other terms: “n-of-1”

Filters: completed studies, studies with results

2021-2023
