# Supplementary material for: A methodological review of randomised n-of-1 trials
Source: Trials. 2024 Apr 16;25:263. doi: 10.1186/s13063-024-08100-1 (PMC11020886; doi:10.1186/s13063-024-08100-1)
Supplement: Supplementary file 3 — Additional file 3. Included Studies. This file contains references to the studies included in the review [file 13063_2024_8100_MOESM3_ESM.docx]

Included studies:

1. Bech AP, Wetzels JF, Groenewoud H, Nijenhuis T. The use of N-of-1 trials to individualize treatment in patients with renal magnesium wasting. American Journal of Kidney Diseases. 2019;73(2):288–90.

2. Benhamou PY, Lablanche S, Vambergue A, Doron M, Franc S, Charpentier G. Patients with highly unstable type 1 diabetes eligible for islet transplantation can be managed with a closed-loop insulin delivery system: a series of N-of-1 randomized controlled trials. Diabetes, Obesity and Metabolism. 2021;23(1):186–94.

3. Brannon EE, Cushing CC, Walters RW, Crick C, Noser AE, Mullins LL. Goal feedback from whom? A physical activity intervention using an N-of-1 RCT. Psychology & health. 2018;33(6):701–12.

4. Ennis JD, Chari VR, Graham A, Harvey DT, Ho E, Nesathurai S. Poster 66 Levodopa/Carbidopa to Improve Motor Recovery After Brain Tumor Excision: An N-of-1 Case Report. PM&R. 2012 Oct;4:S211–S211.

5. Chen Y ming, Deng J min, Wen Y, Chen B, Hou J tao, Peng B, et al. Modified Sijunzi decoction in the treatment of ulcerative colitis in the remission phase: study protocol for a series of N-of-1 double-blind, randomised controlled trials. Trials. 2020 Dec;21(1):396.

6. Cushing C. N-OF-1 RCT FOR DEVELOPING A CONTROL THEORY SMS INTERVENTION FOR ADOLESCENT PHYSICAL ACTIVITY. In: ANNALS OF BEHAVIORAL MEDICINE. SPRINGER 233 SPRING ST, NEW YORK, NY 10013 USA; 2015. p. S50–S50.

7. Ferreira JJ, Mestre T, Guedes LC, Coelho M, Rosa MM, Santos AT, et al. Espresso Coffee for the Treatment of Somnolence in Parkinson’s Disease: Results of n-of-1 Trials. Front Neurol [Internet]. 2016 Mar 8 [cited 2022 Nov 23];7. Available from: http://journal.frontiersin.org/Article/10.3389/fneur.2016.00027/abstract

8. Frost KL, Carey JR, Broback TW, Carlson NL, Daggett CA, Dalbec MM, et al. N-of-1 trial in person with pontine stroke receiving repetitive transcranial magnetic stimulation to improve hand function. Journal of neuroimaging in psychiatry & neurology. 2017;2(2):36.

9. Germini F, Coerezza A, Andreinetti L, Nobili A, Rossi PD, Mari D, et al. N-of-1 Randomized Trials of Ultra-Micronized Palmitoylethanolamide in Older Patients with Chronic Pain. Drugs Aging. 2017 Dec;34(12):941–52.

10. Gurfinkel R, Leushner J, Paul TL, Mahon JL. Use of an N-of-1 Trial to Assess Levothyroxine Intolerance in a Patient with Hypothyroidism. Canadian Journal of Diabetes. 2013 Oct;37:S24–5.

11. Gursli S, Sandvik L, Bakkeheim E, Skrede B, Stuge B. Evaluation of a novel technique in airway clearance therapy – Specific Cough Technique (SCT) in cystic fibrosis: A pilot study of a series of N-of-1 randomised controlled trials. SAGE Open Medicine. 2017 Dec;5:205031211769750.

12. Hsia CC, Mahon JL, Seitelbach M, Chia J, Zou G, Chin-Yee IH. Use of n-of-1 (single patient) trials to assess the effect of age of transfused blood on health-related quality of life in transfusion-dependent patients: AGE OF BLOOD N-OF-1 TRIALS. Transfusion. 2016 May;56(5):1192–200.

13. Huang H, Yang P, Wang J, Wu Y, Zi S, Tang J, et al. Investigation into the Individualized Treatment of Traditional Chinese Medicine through a Series of N-of-1 Trials. Evidence-Based Complementary and Alternative Medicine. 2018;2018:1–11.

14. Huang H, Yang P, Xue J, Tang J, Ding L, Ma Y, et al. Evaluating the Individualized Treatment of Traditional Chinese Medicine: A Pilot Study of N-of-1 Trials. Evidence-Based Complementary and Alternative Medicine. 2014;2014:1–10.

15. Hyam JA, Pereira EAC, McCulloch P, Javed S, Plaha P, Mooney L, et al. Implementing novel trial methods to evaluate surgery for essential tremor. British Journal of Neurosurgery. 2015 May 4;29(3):334–9.

16. Janssen DAW, Hoogenboom T, Heesakkers JPFA. Novel and feasible methodology to obtain LE1 evidence for BPS treatment. Implementing the combined N-of-1 trial design. European Urology Supplements. 2019 Mar;18(1):e1828.

17. Jensen EA, Zhang H, Feng R, Dysart K, Nilan K, Munson DA, et al. Individualising care in severe bronchopulmonary dysplasia: a series of N-of-1 trials comparing transpyloric and gastric feeding. Arch Dis Child Fetal Neonatal Ed. 2020 Jul;105(4):399–404.

18. Joy TR, Monjed A, Zou GY, Hegele RA, McDonald CG, Mahon JL. N-of-1 (Single-Patient) Trials for Statin-Related Myalgia. Ann Intern Med. 2014;160(5):301–10.

19. Kronish IM, Cheung YK, Julian J, Parsons F, Lee J, Yoon S, et al. Clinical Usefulness of Bright White Light Therapy for Depressive Symptoms in Cancer Survivors: Results from a Series of Personalized (N-of-1) Trials. Healthcare. 2019 Dec 30;8(1):10.

20. Lampert A, Tesarz J, Volkert AK, Wesche D, Wild B, Schwab M, et al. An N-of-1 Trial as an Individualized Withdrawal Treatment Approach to Psychological Methylphenidate Dependence. Psychotherapy and psychosomatics. 2014;83(6):379–81.

21. Lee S, Lim N, Choi SM, Kim S. Validation study of Kim’s Sham needle by measuring facial temperature: an N-of-1 randomized double-blind placebo-controlled clinical trial. Evidence-Based Complementary and Alternative Medicine. 2012;2012.

22. Lipka AF, Vrinten C, van Zwet EW, Schimmel KJ, Cornel MC, Kuijpers MR, et al. Ephedrine treatment for autoimmune myasthenia gravis. Neuromuscular Disorders. 2017;27(3):259–65.

23. Marcucci M, Germini F, Coerezza A, Andreinetti L, Bellintani L, Nobili A, et al. Efficacy of ultra-micronized palmitoylethanolamide (um-PEA) in geriatric patients with chronic pain: study protocol for a series of N-of-1 randomized trials. Trials. 2016;17(1):1–12.

24. McGarry ME, Illek B, Ly NP, Zlock L, Olshansky S, Moreno C, et al. In vivo and in vitro ivacaftor response in cystic fibrosis patients with residual CFTR function: N-of-1 studies. Pediatric pulmonology. 2017;52(4):472–9.

25. Nikles J, O’Sullivan JD, Mitchell GK, Smith SS, McGree JM, Senior H, et al. Protocol: Using N-of-1 tests to identify responders to melatonin for sleep disturbance in Parkinson’s disease. Contemporary Clinical Trials Communications. 2019;15:100397.

26. Nikles J, Mitchell GK, Hardy J, Agar M, Senior H, Carmont SA, et al. Testing pilocarpine drops for dry mouth in advanced cancer using n-of-1 trials: A feasibility study. Palliative Medicine. 2015;29(10):967–74.

27. Ong KS, Carlin JB, Fahey M, Freeman JL, Scheffer IE, Gillam L, et al. Protocol for a single patient therapy plan: A randomised, double-blind, placebo-controlled N-of-1 trial to assess the efficacy of cannabidiol in patients with intractable epilepsy. Journal of paediatrics and child health. 2020;56(12):1918–23.

28. Persson MS, Stocks J, Sarmanova A, Fernandes G, Walsh DA, Doherty M, et al. Individual responses to topical ibuprofen gel or capsaicin cream for painful knee osteoarthritis: a series of n-of-1 trials. Rheumatology. 2021;60(5):2231–7.

29. Riggare S, Unruh KT, Sturr J, Domingos J, Stamford JA, Svenningsson P, et al. Patient-driven N-of-1 in Parkinson’s Disease. Methods Inf Med. 2017 Jan;56(S 1):e123–8.

30. Roustit M, Giai J, Gaget O, Khouri C, Mouhib M, Lotito A, et al. On-Demand Sildenafil as a Treatment for Raynaud Phenomenon: A Series of *n* -of-1 Trials. Ann Intern Med. 2018 Nov 20;169(10):694.

31. Samuel JP, Samuels JA, Brooks LE, Bell CS, Pedroza C, Molony DA, et al. Comparative effectiveness of antihypertensive treatment for older children with primary hypertension: study protocol for a series of n-of-1 randomized trials. Trials. 2016;17(1):1–9.

32. Santos C, Weaver DF. Topically applied linoleic/linolenic acid for chronic migraine. Journal of Clinical Neuroscience. 2018;58:200–1.

33. Seitelbach M, Chin-Yee IH, Kinney J, Chia J, Ormond K, Mahon J, et al. Age of Blood Does Not Affect Quality of Life and Hemoglobin: N–of- 1 Trials in Transfusion-Dependent Patients. Blood. 2011;118(21):3369.

34. Senior HE, McKinlay L, Nikles J, Schluter PJ, Carmont SA, Waugh MC, et al. Central nervous system stimulants for secondary attention deficit-hyperactivity disorder after paediatric traumatic brain injury: a rationale and protocol for single patient (n-of-1) multiple cross-over trials. BMC pediatrics. 2013;13(1):1–7.

35. Senior HE, Mitchell GK, Nikles J, Carmont SA, Schluter PJ, Currow DC, et al. Using aggregated single patient (N-of-1) trials to determine the effectiveness of psychostimulants to reduce fatigue in advanced cancer patients: a rationale and protocol. BMC Palliative Care. 2013;12(1):1–6.

36. Sierra-Arango F, Castaño DM, Forero JD, Pérez-Riveros ED, Ardila Duarte G, Botero ML, et al. A Randomized Placebo-Controlled *N* -of-1 Trial: The Effect of Proton Pump Inhibitor in the Management of Gastroesophageal Reflux Disease. Canadian Journal of Gastroenterology and Hepatology. 2019 Dec 18;2019:1–9.

37. Smith JH, Potter JL, Robblee JV. What Is the Optimal Placebo for Clinical Trials of Nerve Blocks in Headache?: An N-of-1 Study of Lactated Ringer’s. Headache: The Journal of Head and Face Pain. 2019;59(1):108–9.

38. Sniehotta FF, Presseau J, Hobbs N, Araújo-Soares V. Testing self-regulation interventions to increase walking using factorial randomized N-of-1 trials. Health Psychology. 2012;31(6):733.

39. Stunnenberg BC, Raaphorst J, Groenewoud HM, Statland JM, Griggs RC, Woertman W, et al. Effect of Mexiletine on Muscle Stiffness in Patients With Nondystrophic Myotonia Evaluated Using Aggregated N-of-1 Trials. JAMA. 2018 Dec 11;320(22):2344.

40. Sun M, Chai L, Lu F, Zhao Y, Li Q, Cui B, et al. Efficacy and Safety of Ginkgo Biloba Pills for Coronary Heart Disease with Impaired Glucose Regulation: Study Protocol for a Series of *N* -of-1 Randomized, Double-Blind, Placebo-Controlled Trials. Evidence-Based Complementary and Alternative Medicine. 2018 Oct 14;2018:1–8.

41. Taye I, Bradbury J, Grace S, Avila C. Probiotics for pain of osteoarthritis; An N-of-1 trial of individual effects. Complementary Therapies in Medicine. 2020 Nov;54:102548.

42. Tison F, Nègre-Pagès L, Meissner WG, Dupouy S, Li Q, Thiolat ML, et al. Simvastatin decreases levodopa-induced dyskinesia in monkeys, but not in a randomized, placebo-controlled, multiple cross-over (“n-of-1”) exploratory trial of simvastatin against levodopa-induced dyskinesia in Parkinson’s disease patients. Parkinsonism & related disorders. 2013;19(4):416–21.

43. Tsiormpatzis S. Effects of Shiatsu on the Health-Related Quality of Life of a Person with Secondary Progressive Multiple Sclerosis. 2019;

44. Vrinten C, Lipka AF, van Zwet EW, Schimmel KJ, Cornel MC, Kuijpers MR, et al. Ephedrine as add-on therapy for patients with myasthenia gravis: protocol for a series of randomised, placebo-controlled n-of-1 trials. BMJ open. 2015;5(7):e007863.

45. Weng S, Fan Z, Qiu G, Liu F, Huang L, Li J, et al. Therapeutic efficacy and immunoregulatory effect of Qiangji Jianli Capsule for patients with myasthenia gravis: Study protocol for a series of randomized, controlled N-of-1 trials. Medicine. 2020 Dec 18;99(51):e23679.

46. Wood FA, Howard JP, Finegold JA, Nowbar AN, Thompson DM, Arnold AD, et al. N-of-1 trial of a statin, placebo, or no treatment to assess side effects. New England Journal of Medicine. 2020;383(22):2182–4.

47. Yuhong H, Qian L, Yu L, Yingqiang Z, Yanfen L, Shujing Y, et al. An n-of-1 Trial Service in Clinical Practice: Testing the Effectiveness of Liuwei Dihuang Decoction for Kidney-Yin Deficiency Syndrome. Evidence-Based Complementary and Alternative Medicine. 2013;2013:1–7.

48. Samuel JP, Tyson JE, Green C, Bell CS, Pedroza C, Molony D, et al. Treating hypertension in children with n-of-1 trials. Pediatrics. 2019;143(4).

49. Ma Y., Fu Y., Tian Y., Gou W., Miao Z., Yang M., et al. Individual postprandial glycemic responses to diet in n-of-1 trials: Westlake N-of-1 trials for macronutrient intake (WE-MACNUTR). J Nutr. 2021;151(10):3158–67.

50. Stunnenberg B.C., Merkus E.C., Raaphorst J., Saris C.G.J., Groenewoud H., Statland J., et al. N-of-1 trial of salbutamol in hyperkalaemic periodic paralysis. J Neurol Neurosurg Psychiatry. 2021;92(12):1352–3.

51. Muller A.R., Zinkstok J.R., Rommelse N.N.J., van de Ven P.M., Roes K.C.B., Wijburg F.A., et al. Methylphenidate for attention-deficit/hyperactivity disorder in patients with Smith-Magenis syndrome: protocol for a series of N-of-1 trials. Orphanet J Rare Dis. 2021;16(1):380.

52. Janssen Daalen J.M., Meinders M.J., Giardina F., Roes K.C.B., Stunnenberg B.C., Mathur S., et al. Multiple N-of-1 trials to investigate hypoxia therapy in Parkinson’s disease: study rationale and protocol. BMC Neurol. 2022;22(1):262.

53. Bashford G., Tan S.X., McGree J., Murdoch V., Nikles J. Comparing pregabalin and gabapentin for persistent neuropathic pain: A protocol for a pilot N-of-1 trial series. Contemp Clin Trials Comm. 2021;24((Bashford) Department of Rehabilitation Medicine, Port Kembla Hospital, Wollongong, Australia):100852.

54. Mitsumoto H., Cheung K., Oskarsson B., Andrews H.F., Jang G.E., Andrews J.A., et al. Randomized double-blind personalized N-of-1 clinical trial to test the safety and potential efficacy of TJ-68 for treating muscle cramps in amyotrophic lateral sclerosis (ALS): study protocol for a TJ-68 trial. Trials. 2023;24(1):449.

55. Kaplan H.C., Opipari-Arrigan L., Yang J., Schmid C.H., Schuler C.L., Saeed S.A., et al. Personalized Research on Diet in Ulcerative Colitis and Crohn’s Disease: A Series of N-of-1 Diet Trials. Am J Gastroenterol. 2022;117(6):902–17.

56. Zi S., Huang H., Yang P., Xu M., Wu Y., Wang Z., et al. Evaluating the Effects of Heat-Clearing Traditional Chinese Medicine in Stable Bronchiectasis by a Series of N-of-1 Trials. Evid-Based Complement Altern Med. 2022;2022((Zi, Huang, Yang, Xu, Wang, Ge) Yueyang Hospital of Integrated Traditional Chinese and Western Medicine, Shanghai University of Traditional Chinese Medicine, Shanghai 200437, China):6690638.

57. Weng S., Li J., Chen B., He L., Zhong Z., Huang L., et al. Effectiveness of modified Buzhong Yiqi decoction in treating myasthenia gravis: study protocol for a series of N-of-1 trials. Trials. 2022;23(1):365.

58. Ambros G., Valoes R., dos Santos Carregosas A.L., Callegari-Jacques S.M., Forcelini C.M., Fornari F. Gastric Glitch: A New Functional Disease Treated with Buspirone and Prucalopride in an N-of-1 Double-Blind Clinical Trial. Clin Drug Invest. 2023;43(1):75–8.

59. Bapir M, Campagnolo P, Rodriguez-Mateos A, Skene SS, Heiss C. Assessing Variability in Vascular Response to Cocoa With Personal Devices: A Series of Double-Blind Randomized Crossover n-of-1 Trials. Front Nutr [Internet]. 2022 Jun 13 [cited 2024 Mar 14];9. Available from: https://www.frontiersin.org/articles/10.3389/fnut.2022.886597

60. Adler U.C., Adler M.S., Cesar A.D.T., Santos H.D.F., Magalhaes P.A.D.F., Nogueira R.D.S., et al. Homeopathy for Major Depressive Disorder: Protocol for N-of-1 Studies. Comp Med Res. 2023;30(4):332–9.

61. Klotz R., Emile G., Daviet J.-C., De Seze M., Godet J., Urbinelli R., et al. Daily socket comfort in transtibial amputee with a vacuum-assisted suspension system: study protocol of a randomized, multicenter, double-blind multiple N-of-1 trial. BMC Sports Sci Med Rehabil. 2023;15(1):85.

62. Gursli S., Quittner A., Jahnsen R.B., Skrede B., Stuge B., Bakkeheim E. Airway clearance physiotherapy and health-related quality of life in cystic fibrosis - a substudy of a series of n-of-1 randomised controlled trials. J Cyst Fibrosis. 2022;21(Supplement 1):S126.

63. Zhai J, Liu AF, Yu W, Guo T. Baduanjin exercise for chronic non-specific low back pain: protocol for a series of N-of-1 trials. BMJ Open. 2023 Nov 1;13(11):e070703.

64. Dolmage T.E., Goldstein R.S. Assisting Walking in Patients with Chronic Respiratory Disease Using a Powered Exoskeleton: A Series of N-of-1 Clinical Trials. Ann Am Thorac Soc. 2022;19(7):1230–2.

65. Nurmi J, Knittle K, Naughton F, Sutton S, Ginchev T, Khattak F, et al. Biofeedback and Digitalized Motivational Interviewing to Increase Daily Physical Activity: Series of Factorial N-of-1 Randomized Controlled Trials Piloting the Precious App. JMIR Formative Research. 2023 Nov 23;7(1):e34232.

66. Friel CP, Robles PL, Butler M, Pahlevan-Ibrekic C, Duer-Hefele J, Vicari F, et al. Testing Behavior Change Techniques to Increase Physical Activity in Middle-Aged and Older Adults: Protocol for a Randomized Personalized Trial Series. JMIR Research Protocols. 2023 Jun 14;12(1):e43418.

67. Li K, Bauer S, Kenfield S, Lu K, McCulloch C, Steinman M, et al. MP24-04 RANDOMIZED PLACEBO-CONTROLLED N-OF-1 TRIALS OF TAMSULOSIN FOR LOWER URINARY TRACT SYMPTOMS. Journal of Urology. 2023 Apr;209(Supplement 4):e318.

68. Verploegen MF, Wieërs ML, Giardina F, Trepiccione F, Walsh SB, Hoorn EJ, et al. Using an Innovative N-of-1 Trial Approach Testing Efficacy of Salt Supplementation in Gitelman Syndrome. In Orlando, FL, United States: American Society of Nephrology; 2022 [cited 2024 Mar 15]. p. 416. Available from: https://www.asn-online.org/api/download/?file=/education/kidneyweek/archives/KW22Abstracts.pdf

69. Crook N, Adams M, Shorten N, Langdon PE. Does the Well-Being of Individuals with Down Syndrome and Dementia Improve When Using Life Story Books and Rummage Boxes? A Randomized Single Case Series Experiment. Journal of Applied Research in Intellectual Disabilities. 2016;29(1):1–10.

70. Khasnavis T, Torres RJ, Sommerfeld B, Puig JG, Chipkin R, Jinnah HA. A double-blind, placebo-controlled, crossover trial of the selective dopamine D1 receptor antagonist ecopipam in patients with Lesch-Nyhan disease. Molecular Genetics and Metabolism. 2016 Jul 1;118(3):160–6.

71. Muller A., Konigorski S., Meissner C., Fadai T., Warren C.V., Falkenberg I., et al. Study protocol: combined N-of-1 trials to assess open-label placebo treatment for antidepressant discontinuation symptoms [FAB-study]. BMC Psychiatry. 2023;23(1):749.

72. Weill Medical College of Cornell University. N-of-1 Trials for Deprescribing Beta-blockers in HFpEF [Internet]. clinicaltrials.gov; 2023 Aug [cited 2024 Jan 1]. Report No.: NCT04767061. Available from: https://clinicaltrials.gov/study/NCT04767061

73. Samuel JP. Assessing the Feasibility of N-of-1 Trials in Children With Hypertension and Chronic Kidney Disease: a Pilot Study [Internet]. clinicaltrials.gov; 2023 Feb [cited 2024 Jan 1]. Report No.: NCT04591171. Available from: https://clinicaltrials.gov/study/NCT04591171

74. University of Minnesota. Cilostazol for HFpEF (Heart Failure With a Preserved Ejection Fraction) [Internet]. clinicaltrials.gov; 2023 Nov [cited 2024 Jan 1]. Report No.: NCT05126836. Available from: https://clinicaltrials.gov/study/NCT05126836
